# Simultaneous *in vitro* generation of CD8 and CD4 T cells specific to three universal tumor associated antigens of WT1, survivin and TERT and adoptive T cell transfer for the treatment of acute myeloid leukemia

**DOI:** 10.18632/oncotarget.17212

**Published:** 2017-04-19

**Authors:** Hyun-Jung Sohn, Ji Yoon Lee, Hyun-Joo Lee, Dae-Hee Sohn, Hyun-Il Cho, Hee-Je Kim, Tai-Gyu Kim

**Affiliations:** ^1^ Catholic Hematopoietic Stem Cell Bank, The Catholic University of Korea, Seoul, Korea; ^2^ Leukemia Research Institute, Seoul St. Mary`s Hospital, College of Medicine, The Catholic University of Korea, Seoul, Korea; ^3^ Department of Hematology, Catholic Blood and Marrow Transplantation Center, Seoul St. Mary`s Hospital, College of Medicine, The Catholic University of Korea, Seoul, Korea; ^4^ Departments of Microbiology, College of Medicine, The Catholic University of Korea, Seoul, Korea; ^5^ Department of Biomedical Laboratory Science, College of Health Sciences, Sangji University, Wonju, Korea; ^6^ ViGenCell Inc., Seoul, Korea

**Keywords:** Wilms’ tumor protein 1, survivin, telomerase reverse transcriptase, dendritic cells, adoptive T cell therapy

## Abstract

Previously, we found that most patients with acute myeloid leukemia (AML) expressed at least one of the leukemic associated antigens (LAAs) *WT1, survivin* and *TERT*, and different combinations of the three LAAs predicted negative clinical outcomes. Multi-tumor antigen-specific T cells were generated to overcome antigenic variation and may be sufficient to maximize antitumoral effects. To generate triple antigen-specific (Tri)-T cells that recognize three LAAs, dendritic cells (DCs) were transfected with three tumor antigen-encoding RNAs. These DCs were used to stimulate both CD8 and CD4 T cells and to overcome the limitation of known human leukocyte antigen-restricted epitopes. The sum of the antigen-specific T cell frequencies was higher in the Tri-T cells than in the T cells that recognized a single antigen. Furthermore, the Tri-T cells were more effective against leukemic blasts that expressed all three LAAs compared with blasts that expressed one or two LAAs, suggesting a proportional correlation between IFN-γ secretion and LAA expression. Engrafted leukemic blasts in the bone marrow of mice significantly decreased in the presence of Tri-T cells. This technique represents an effective immunotherapeutic strategy in AML.

## INTRODUCTION

Various immunotherapeutic approaches have been developed based on tumor-associated antigens (TAAs), including the adoptive transfer of cytotoxic T lymphocytes (CTLs), direct vaccination, and dendritic cell (DC)-based vaccines. These methods have proven to be effective methods in tumor regression [1–4]. Despite the discovery of TAAs, a major hurdle in cancer immunotherapy has been the lack of antigens shared by patients with common cancers. To circumvent this obstacle, several TAAs have been suggested as universal TAAs that can trigger CTL responses against a broad range of tumor types. These TAAs of hematologic malignancies are similar to those of solid tumors [5–8]. Several TAAs have been identified in patients with acute myeloid leukemia (AML). These include BCL-2, OFA-iLRP, BAGE, FLT3-ITD, PRAME, G250, telomerase reverse transcriptase (*TERT*), proteinase 3, RHAMM, *survivin* (known as *BIRC5*) and Wilms’ tumor protein (*WT1*). These antigens have been shown to trigger CD8^+^ T-cell recognition of tumor cells, and some antigens also induce humoral immune responses [9]. Expression of the genes encoding *WT1*, *survivin* and *TERT* is used as a prognostic marker, and these TAAs have been characterized as powerful tumor antigens in AML [5, 8, 10]. Previously, we reported that most patients with AML express at least one of these antigens, and the combinational expression of more than one antigen strongly predicts poor clinical outcomes [5].

The wild-type *WT1* gene is increased in various types of malignant tumors, such as thyroid, lung, breast, and colorectal cancers [11, 12]. Furthermore, higher levels of wild-type *WT1* have been found in most cases of leukemia, compared with normal bone marrow (BM) or peripheral blood (PB) [13, 14]. Adoptive transfer of *WT1*-specific CD4^+^ and CD8^+^ T cells can induce the sustained remission of refractory AML [15]. The administration of *WT1*-specific CD8^+^ cytotoxic T-cell (CTL) clones after Hematopoietic stem cell transplantation to relapsed or high-risk leukemia patients led to detectable long term maintenance and clinical responses without evidence of on-target toxicity [16]. *Survivin*, a member of the inhibitors of apoptosis (IAP) protein family, is frequently increased in cancer but is rarely detected in normal adult tissues [17]. Overexpressed *survivin* correlates with an apoptosis-resistant phenotype in chronic myeloid leukemia cells. It is significantly increased in CD34^+^CD38^−^ leukemia stem/progenitor cells and predicts poor clinical outcomes in AML [18]. Spontaneous anti-*survivin* T-cell reactivity has been described in cancer patients suffering from a huge range of cancers, including breast and colon cancer, lymphoma, leukemia, and melanoma [19–22]. Most human cells do not have telomerase activity or human *TERT* expression [23–25]. In contrast, a great majority of human tumors exhibit strong telomerase activity [23], express human *TERT* [24–26], and maintain the lengths of their telomeres [27, 28]. Data from both human and murine systems demonstrate that CTLs can recognize peptides derived from *TERT* and kill *TERT*-positive tumor cells [2]. *TERT* RNA-transfected human DCs also stimulated *TERT*-specific CTLs that effectively lysed tumor cells [29].

TAA-specific CD8 and CD4 T cells play a pivotal role in anti-tumor immunity. However, this immune surveillance system can be circumvented by tumorigenic cells that have developed a variety of immune evasion mechanisms, including immune editing, antigenic heterogeneity, and loss of antigens such as human leukocyte antigen (HLA) or TAAs [30, 31], which results in weak T-cell recognition and the incomplete elimination of malignant cells [32–35]. Most tumor cells express high levels of multiple TAAs [36], which accounts for the significantly lower surveillance efficiency of single-antigen specific T cells (Single-T cells) compared with multiple antigen-specific lymphocytes. CTLs specific to several TAAs have a greater ability than Single-T cells to effectively kill leukemic cells because they can compensate if one antigen is lost or edited [37]. Multi-TAA-specific T-cells against myeloid malignancies were generated by stimulation with 15mer peptide libraries of five TAAs (proteinase 3, *WT1*, human neutrophil elastase and melanoma-associated antigen A3) and were able to recognize partially HLA-matched myeloid leukemia blasts [15]. Combination immunotherapy using adoptive T-cell transfer and vaccination on the basis of *TERT* and *survivin* as universal TAAs was performed after autologous stem cell transplantations for myeloma [38]. These multiple antigen-specific-T cells have been generated using antigen-presenting cells loaded with peptides or mixtures of peptides [37, 39, 40].

In this study, T cells capable of recognizing the three universal TAAs *WT-1, survivin* and *TERT* (Tri-T cells) were generated to overcome the limitations of known HLA-restricted epitopes. DCs were electroporated with mRNA so they could present functional antigenic peptides to CTLs. Additionally, this approach simultaneously stimulated the expansion of many antigen-specific CD8+ and CD4+ T cells [41, 42]. The Tri-T cells produce anti-leukemia immune responses, including the appropriate memory and effector T cell phenotypes, against primary myeloblasts, and this paves the way for advanced AML immunotherapy.

## RESULTS

### Viability and antigen expression in human DCs transfected with LAA RNAs

DCs were transfected with complete tumor antigen-coding RNA sequences to overcome the limitations of known HLA-restricted epitopes. When different RNA transfection methods were compared, the electroporation-based nucleofection of DCs, using the Nucleofector X1 program, demonstrated 60% superior transfection efficiency, cell viability, and protein expression compared with other methods (Online Supplementary Figure 1A). This method was selected to generate three tumor antigens-specific T cells. Three tumor antigen-encoding RNAs were separately expressed in transfected DCs (Mock, 0.1–0.4 copies; *WT1*, 1002.7 copies; *survivin*, 7080.5 copies; *TERT*, 166.5 copies; Table 1), which was confirmed by real-time PCR. We also found that, compared with DNA-transfected DCs, RNA-transfected DCs could induce significantly higher CTL responses (data not shown). The RNA concentration optimal for DC viability and protein expression was established as 20 μg (Online Supplementary Figure 1B) and was used throughout the study.

**Table 1:** *WT1, survivin, TERT* expression in DCs after transfer of antigen *in vitro* transcribed mRNA

Cells	mRNA Copies/ 10^5^ beta-Actinz=2^ΔCtNORMx10^5^		
	*WT1*	*survivin*	*TERT*
Mock-DCs	0.1	0.4	0.4
DCs/WT1 RNA	1002.7	0.4	0.4
DCs/survivin RNA	0.2	7080.5	0.4
DCs/TERT RNA	0.2	0.4	166.5

*WT1, survivin, TERT* mRNA expression levels were quantified using a quantitative real-time PCR assay.

### *In vitro* generation of Tri-T cells is similar to that of Single-T cells

**Figure 1:**
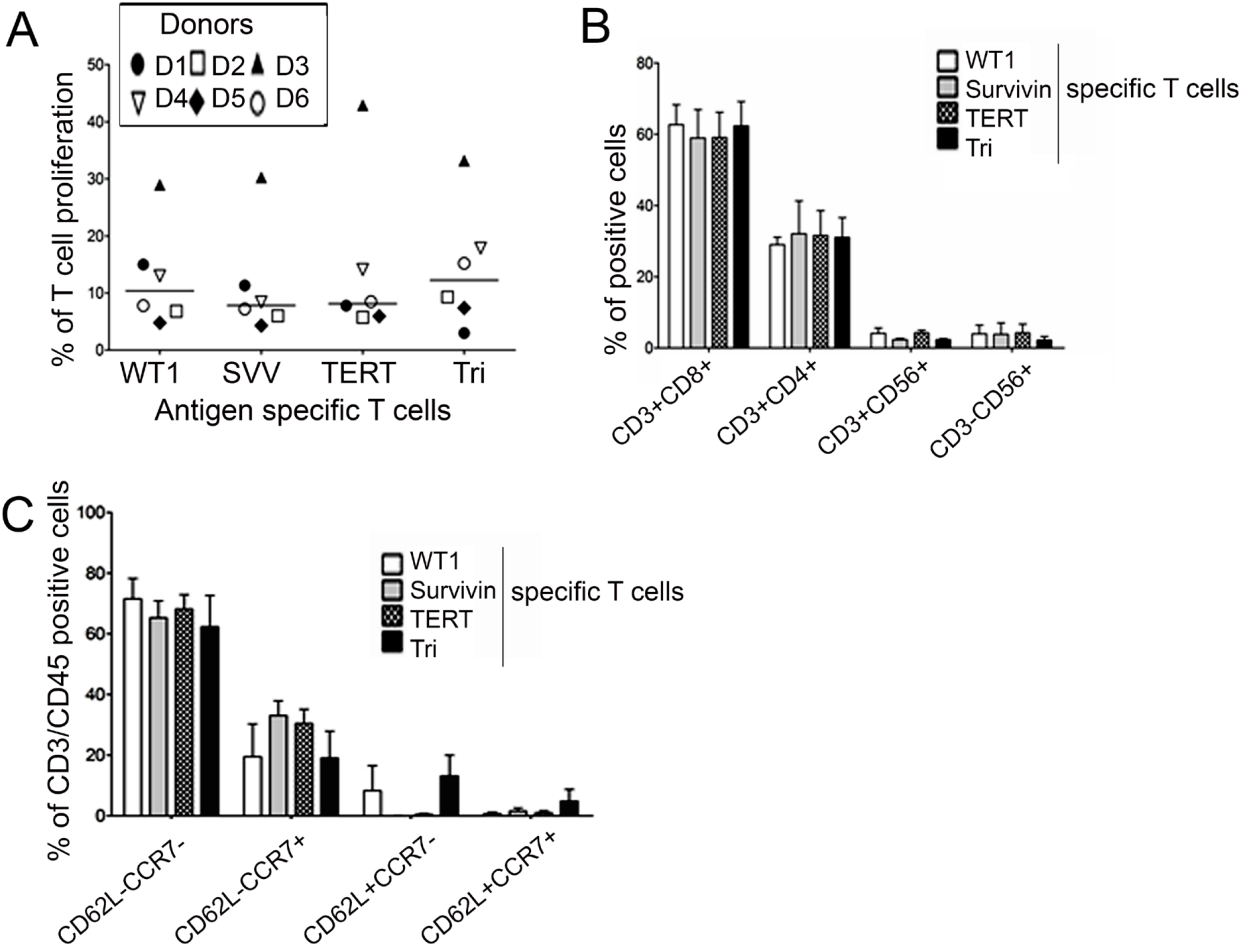
Generation of single antigen specific (Single)- and triple antigen specific (Tri)-T cells against *WT1, survivin*, and *TERT* Tri-T cells were generated using DCs with electroporated IVT mRNA (*WT1* and *survivin* and *TERT*) in the presence of IL-2 and IL-15. At day 21, T cells were characterized by FACS and ELISpot assays. **(A)** Single-T cells generated from the same donors served as controls. **(B, C)** There was no difference in cell proliferation or phenotypes of Single- and Tri-T cells in lymphocytes or effector/central memory cells.

To generate Tri-T cells that recognize all three LAAs, PBMCs from six healthy donors were co-cultured for 21 days in the presence of IL-2 and IL-15 with DCs transfected with RNA that was transcribed *in vitro* from full-length human *WT1, survivin*, and *TERT* genes. DCs expressing whole LAA antigens successfully stimulated Tri-T cells in all donors who had different HLA types. Overwhelmingly, Tri-T cells could recognize single antigens as well as triple LAAs, indicating superior functional activity. The generated Tri- and Single-T cells had no difference in cell proliferation (Figure 1A) or in their responses to single mRNA-transfected DCs. Tri- and Single-T cells cultured with single LAA-transfected DCs responded to those DCs but not to the other LAA-transfected DCs. The frequencies of CD4^+^, NK, and NKT cells was lower than that of CD8^+^ cells; however, there was no difference between Single-T cells and Tri-T cells in these categories of immune cells (CD8^+^ cells: *WT1*, 62.7% ± 9.7%; *survivin*, 58.9% ± 13.7%; *TERT*, 59.1% ± 12.3%; Tri, 62.3% ± 11.8%; CD4^+^ cells: *WT1*, 29% ± 3.6%; *survivin*, 32% ± 16%; *TERT*, 31.5% ± 11%; Tri, 31% ± 9.6%; NK^+^ cells: *WT1*, 3.9% ± 4.2%; *survivin*, 3.7% ± 5.5%; *TERT*, 4.1% ± 4.3%; Tri, 2.1% ± 1.7%; NKT^+^ cells: *WT1*, 4.1% ± 2.5%; *survivin*, 2.3% ± 0.5%; *TERT*, 4.1% ± 1.4%; Tri, 2.3% ± 0.4%; Figure 1B). The percentage of CD62L^−^CCR7^−^ effector memory cells was higher (66.8% ± 11%) than that of other memory cells, including CD62L^−^CCR7^+^, CD62^+^CCR7^−^, and CD62L^+^CCR7^+^ (25.4% ± 13%, 5.4% ± 9.7%, and 1.9% ± 3.4%, respectively; Figure 1C). These results indicate that Tri-T cells were generated with efficiency comparable to that of Single-T cells, and Tri-T cells were specific to multiple LAAs, which allows for the killing of tumor cells expressing either one or all of the tested LAAs, regardless of the expression mode.

### Tri-T cells can induce higher immune responses to three LAAs than single-T cells

**Figure 2:**
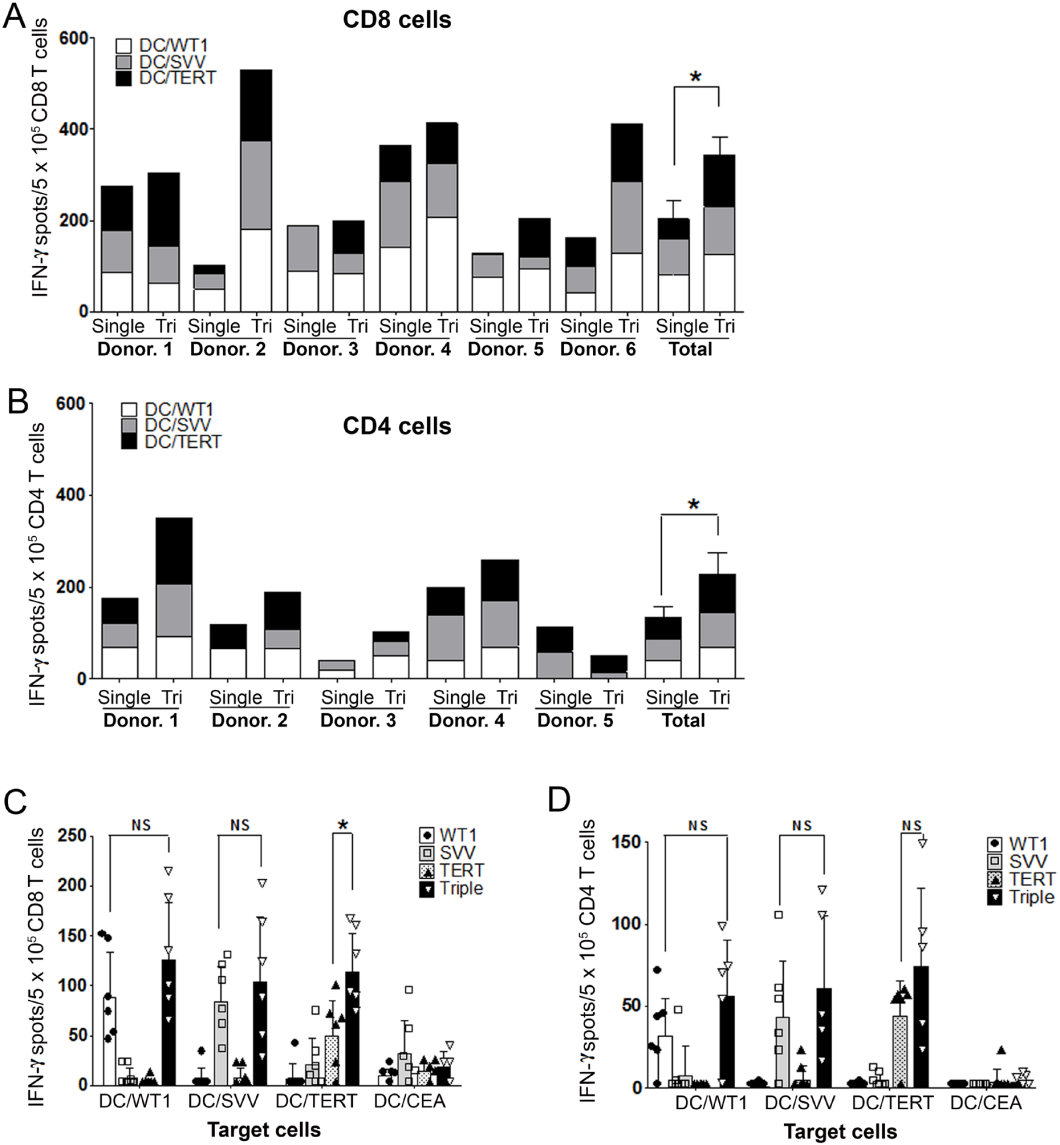
Comparison of Single- versus Tri-T cells against *WT1, survivin*, and *TERT* **(A)** ELISpot assays were performed using purified CD8 T cells. **(B)** ELISpot assays were performed using purified CD4 T cells. **(C)** *TERT* increased the most in Tri-CD8 T cells and led to a dramatic increase in Tri-CD8 T cell activation. **(D)** No significant difference was detected in Tri-CD4 T cells. Data shown represent the means ± SE of six donors. (**, *P* < 0.01).

To examine the immune response of Tri-T cells, IFN-γ secretion was analyzed by ELISpot in CD8^+^ (Tri- or Single-CD8 T) and CD4^+^ (Tri- or Single-CD4 T) cells sorted from Tri- or Single-T populations. Interestingly, IFN-γ production dramatically increased in Tri-CD8 T cells, compared with Single-CD8 T cells, suggesting that the response of Tri-CD8 T cells to certain tumor antigens can be stronger than that of Single-CD8 T cells (Figure 2A), which may miss or react weakly to a specific subset of leukemic cells. IFN-γ production by Tri-CD8 T cells increased most dramatically in donors 2, 3, 5 and 6. This increase results from upregulated *TERT* in most donors except no. 4. (IFN-γ spot in *TERT*; Single-CD8 T cells, 50.2± 34.4; Tri-CD8 T cells, 113.8± 38.3; Figure 2C). These results suggest that Tri-CD8 T cells may simultaneously strengthen a weak response to *TERT* by supportive factors from other tumor antigens and respond to other tumor antigens. *WT1* and *survivin* also increased, but not significantly. Tri-CD4 T cells also produced higher levels of IFN-γ than Single-CD4 T cells, except donor no. 5 (Figure 2B). Tri-CD4 T cells had similar increases in *TERT, survivin*, and *WT1* (Figure 2D). Tri-T cells generated from normal PBMCs displayed differences in their ability to recognize LAAs, indicating individual variability (Figure 2A, 2B and online Supplementary Table 1). Despite this variability, results clearly showed the high production of IFN-γ in Tri-T cells, suggesting that Tri-T cells can be generated in large quantities by using antigen combinations and may be beneficial to AML treatment.

### The proportion of T cells specificity for three LAAs in Tri-T cells

**Figure 3:**
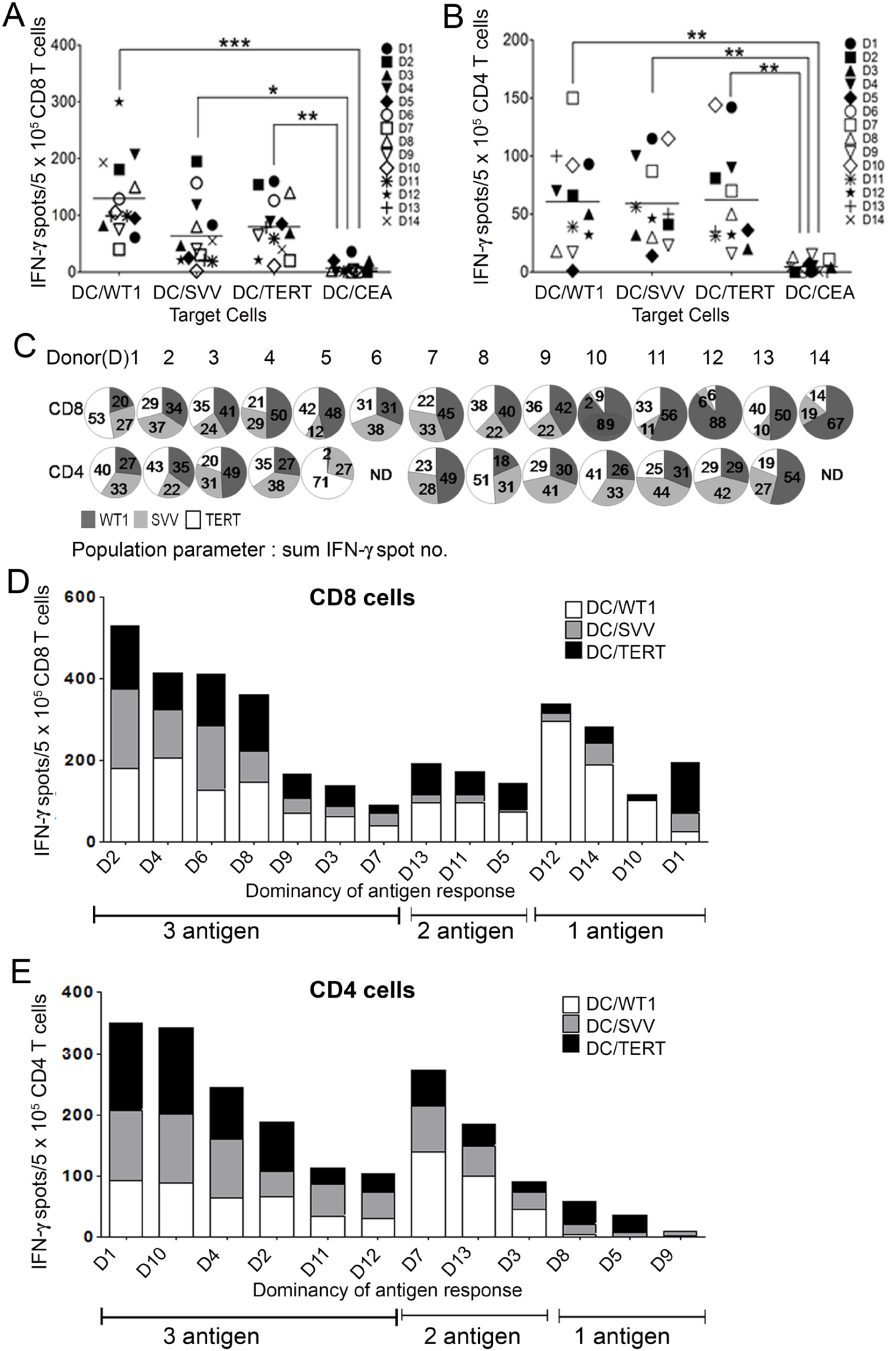
The proportions of T cells specific for *WT1, survivin* and *TERT* antigens in Tri-T cells from 14 individual donors In CD8 T cells **(A)** and CD4 T cells **(B)**, the total specificity for *WT1, TERT*, and *survivin* was consistent, regardless of individual specificity **(A, B)**. Tri-CD8 T cells and Tri-CD4 T cells were most specific to *WT1* and *TERT*, respectively. **(C)**. A diagram shows individual variability in generated Tri-T cells. **(D)** and **(E)** depict IFN-γ production in Tri-CD8 and CD4 T cells, revealing a broad distribution for the three LAAs. (***, *P* < 0.001, **, *P* < 0.01, *, *P* < 0.05)

To examine the specificity of Tri-T cells based on individual donors, IFN-γ-secreting CD8^+^ and CD4^+^ T cells were sorted from the total Tri-T population and analyzed by ELISpot. CD8^+^ and CD4^+^ cells produced more IFN-γ when Tri-T cells were stimulated with single antigen-expressing DCs compared with the control CEA group (Figure 3A and 3B). Tri-T cells stimulated by normal PBMCs differed in their ability to recognize LAAs, indicating individual variability (Figure 3C, Table 2). Because three LAAs were used, 45% was determined to be the limit for a majority. Based on the proportions of Tri-CD8 T cells specific for *WT1, survivin* and *TERT*, antigen specificity for *WT1* appeared to be dominant, as it was over 45% in the 8 donors (no. 4, 5, 7, 10, 11, 12, 13, 14, Figure 3C and 3D). Although TERT was the most prominent in one donor (no. 1), no donor stimulated a majority of Tri-CD8 T cells specific to *survivin*. Tri-CD8 T cells showed specificity to *WT1, TERT*, and *survivin*, in descending order (Figure 3A and 3D). In Tri-CD4 T cells, specificity for *WT1* and *TERT* was dominant in 3 donors (no. 3, 7, 13) and 2 donors (no. 5, 8), respectively (Figure 3C and 3E). Similar to what was observed in Tri-CD8 T cells; Tri-CD4 T cells were less specific to *survivin*. Based on data from human samples, these proportions of antigen specificity were not evenly distributed among generated Tri-T cells. Most Tri-T cells had balanced distribution of the three LAAs (Tri-CD8 T cells, no. 2, 3, 4, 6, 8, 9; Tri-CD4 T cells, no. 1, 2, 4, 10, 11, 12), but some Tri-CD8 or CD4 T cells were mainly stimulated by *WT1* and *TERT* (Tri-CD8 T cells, no. 5, 7, 11, 13; Tri-CD4 T cells, no. 3, 7, 13) or only one LAA (Tri-CD8 T cells, no. 1, 10, 12, 14; Tri-CD4 T cells, no. 5, 8, 9) (Figure 3C, 3D and 3E). Additionally, 2 donors (no. 6, 14) failed to generate Tri-T cells, and ELISpot assays were not performed (Figure 3C). Total antigen specificity for *WT1, TERT*, and *survivin* was stable, regardless of individual specificity. These results can be very informative in generating multi-leukemia antigen-specific T cells and indicate the successful generation of Tri-T cells that recognize 3 LAAs.

**Table 2:** Recognition of individual target antigens in IFN- γ EliSpot assay by Tri- CD8 and CD4 T cells

no.	HLA-A*02:01	IFN-γ spots no./5×10^5^ CD8 T cells				IFN-γ spots no. /5×10^5^ CD4 T cells			
		WT1	SVV	TERT	CEA	WT1	SVV	TERT	CEA
N1	+	61	83	160	36	93	115	142	0
N2	+	181	195	154	0	66	41	81	0
N3	+	82	47	69	20	50	32	20	4
N4	+	207	118	89	0	70	100	90	5
N5	+	95	25	85	20	1	14	36	7
N6	+	129	157	126	0	ND	ND	ND	ND
N7	+	40	30	20	0	150	87	70	11
N8	+	150	80	140	3	18	30	50	13
N9	+	75	40	65	4	17	23	16	15
N10	+	105	2	11	2	92	115	144	3
N11	+	99	19	59	1	39	56	31	4
N12	+	300	21	21	4	32	46	32	2
N13	−	99	21	79	2	100	50	35	0
N14	+	193	55	40	2	ND	ND	ND	ND
Median(IQR; 25–75%)		97(56–158)	35(21–92)	67(21–130)	0(0–0)	44.5(13–93)	43.5(21–90)	35.5(19–144)	0(0–0)

IQR: Interquartile range, ND: Not done.

### Immune responses of Tri-T cells against AML patient-derived blasts

**Figure 4:**
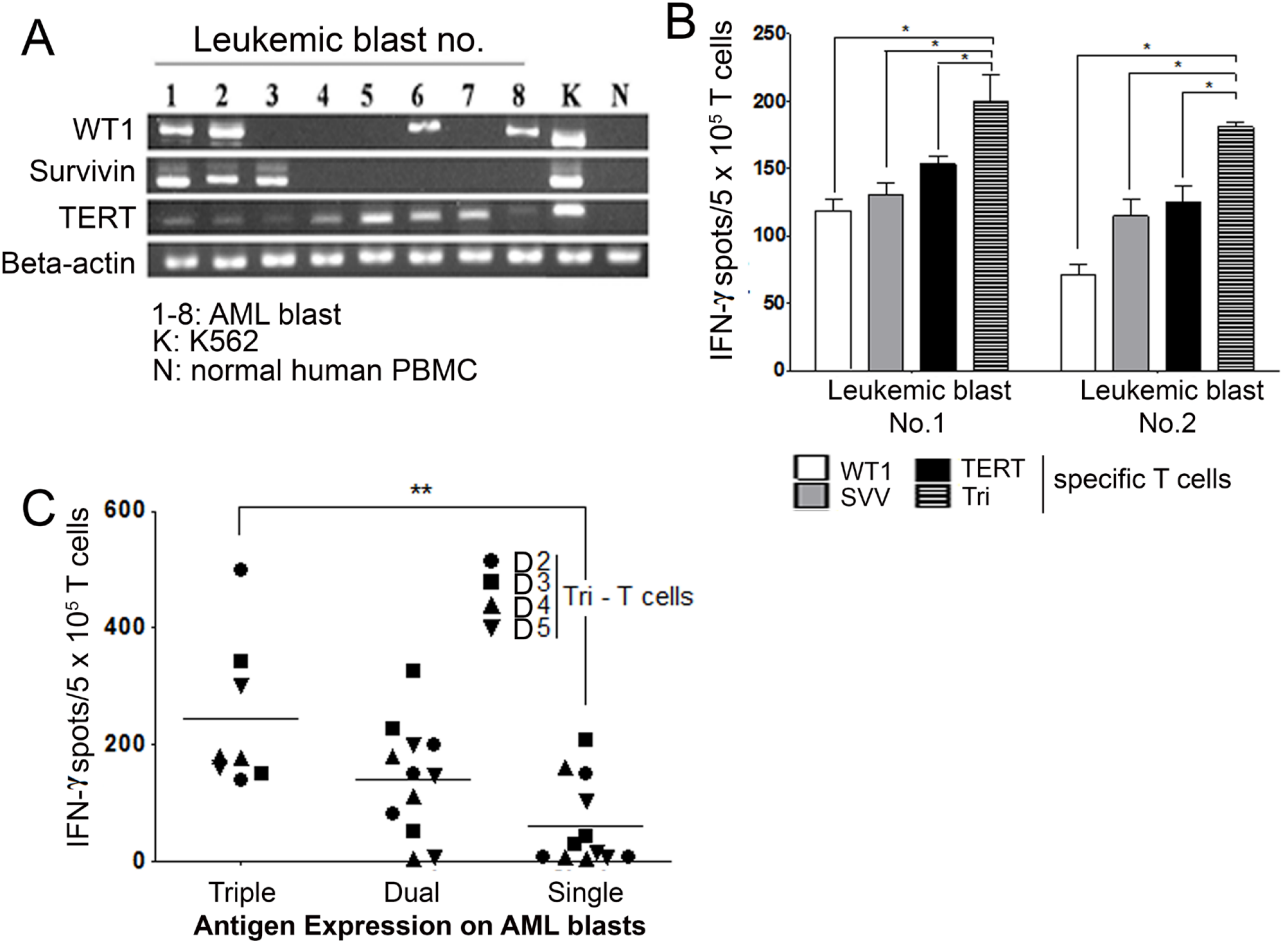
Tri-T cells can function as effector cells against AML blasts **(A)** RT-PCR analysis of the expression of *WT1, survivin* or *TERT* in AML blasts from eight patients. IFN-γ production by Tri-T cells depends on antigen combinations. **(B)** IFN-γ production by Single- and Tri-T cells co-cultured with AML blasts no. 1 and no. 2 in ELISpot assay. Tri-T cells produced more IFN-γ compared with Single-T cells. (*, *P* <0.05) **(C)** Production of IFN-γ by Tri-T cells co-cultured with single, dual or triple tumor antigen-expressing AML blasts in ELISpot assay. Values shown are the averages of experiments performed in triplicate. Bars represent the means ± SE of all experiments. (**, *P* <0.01 vs. AML blasts).

Next, we performed the ELISpot assay to analyze the immune response of Tri-T cells in myeloblasts isolated from AML patients. Previously, we have shown that 90% of AML patients express at least one of the LAAs *WT1, survivin*, and *TERT* and that their bone marrow (BM) levels are 13.8–86.7-fold higher compared with normal donors [5]. Here, we also detected high expression of these antigens in eight patients with AML. Among these patients, two (patients no. 1 and 2) expressed all three LAAs, three (patients no. 3, 6, and 8) expressed two LAAs (*survivin + TERT* and *WT1 + TERT*), and three patients (no. 4, 5, 7) expressed single antigens (Figure 4A). K562 cells expressing all three antigens were used as a positive control [42–44]. To determine the activity of Tri-T cells against AML primary blasts, the ELISpot assay was first performed using blasts from AML patients 1 and 2, who expressed all 3 LAAs. As shown in Figure 4B, in response to blasts from patient 1, IFN-γ production by Tri-T cells significantly increased compared with Single-T cells (Figure 4B). Next, we examined Tri-T cell function in terms of LAA expression. Tri-T cells induced with single LAA-expressing myeloblasts (no. 4, 5, 8) produced the least IFN-γ, and Tri-T cells induced with triple LAA-expressing blasts produced the most IFN-γ (no. 1, 2) (Figure 4C). IFN-γ levels were higher in dual LAA-expressing blast-stimulated cells than in single LAA-expressing blasts-stimulated cells, but the difference was not statistically significant. These data indicates conspicuous function of Tri-T cells against 3 LAA-expressing myeloblasts.

### Anti-leukemia effects of Tri-T cells in NSG mouse model

**Figure 5:**
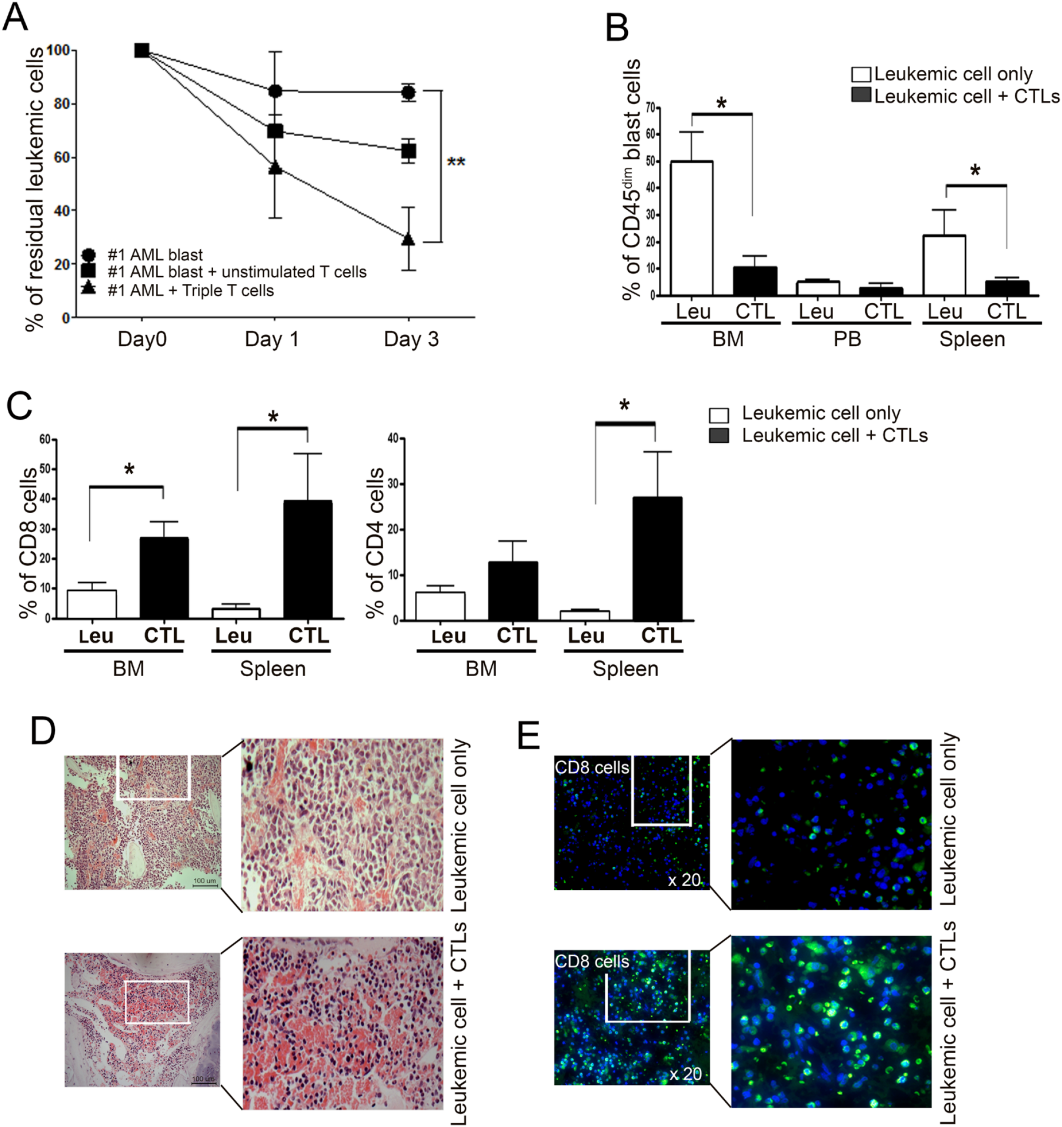
A large percentage of residual leukemic cells associated with Tri-T cells and xenograft leukemic mice are CD45^dim^ blasts **(A)** Recognition of primary AML blasts by Tri-T cells. Co-culture of Tri-T cells with HLA-A*02:01-matched AML blasts expressing *WT1*, *survivin*, and *TERT*. The residual leukemia blasts were calculated and expressed as a percentage of 0 day CD33^+^CD34^+^ “AML blast” group, which was calculated as 100%. Values shown are the averages of experiments performed in triplicate. Bars represent the means ± SE of all experiments. (**, *P* <0.01 vs. AML blasts) **(B)** FACS analysis reveals high levels of CD45^dim^ blasts in xenograft mouse BM and spleen. **(C)** Increased CD8 and CD4 cells in mice injected with Tri-T cells. **(D)** H&E staining showed a striking difference in leukemic blasts (upper) and lymphocytes (lower) in mouse BM. Scale bar = 100 μm. **(E)** Immunocytochemistry data shows CD8 T cell infiltration in BM. DAPI (blue) for nuclear staining, FITC (green) in enlarged image shows CD8 T cells after injection of Tri-T cells. 20 x magnifications.

Next, we investigated the anti-leukemic effect of Tri-T cells against primary AML blasts by calculating residual leukemic cells after co-culturing AML blasts with Tri-T lymphocytes. Tri-T cells generated from donors 1, 4, and 8 showed significant anti-leukemic effects compared with the control group without Tri-T cells and eradicated leukemic blasts derived from patients 1 and 2 (Figure 5A). To investigate whether infiltrating Tri-T cells could eliminate human myeloblasts *in vivo*, primary leukemic mononuclear cells isolated from patients with AML were engrafted into irradiated immunodeficient NSG mice by tail vein injection. After 5 weeks, the engrafted AML blasts were analyzed for the expression of the leukocyte antigen CD45. Human AML blasts have no expression or low expression of CD45 (CD45^dim/−^ phenotype), whereas normal lymphocytes strongly express CD45 (CD45^bright^ phenotype) [45]. Thus, the fraction of CD45^dim^ cells was used as an indication of leukemic blast engraftment and elimination by injected Tri-T cells in NSG mice. At five weeks after blast injection, FACS data showed a decrease in the CD45^dim^ cell population in the Tri-T cell-treated mice compared with Tri-T-untreated mice (in the BM, 10.30% ± 5.43% versus 49.03% ± 11.68%; in peripheral blood, 2.52% ± 2.00% versus 4.52% ± 1.42%; in the spleen, 5.01% ± 2.38% versus 22.14% ± 9.47%; Figure 5B). In contrast, the levels of CD8^+^ and CD4^+^ cells in the BM and spleen were higher in AML mice treated with Tri-T cell infusion than in the AML mice that were not treated with Tri-T cell infusion (for CD8^+^ cells in the BM, 26.69% ± 2.10% versus 7.71% ± 4.18%; in the spleen, 38.65% ± 16.47% versus 3.06% ± 1.75%; for CD4^+^ cells in the BM, 12.83% ± 4.54% versus 5.73% ± 1.81%; in the spleen, 26.85% ± 10.01% versus 1.83% ± 0.45%; Figure 5C), indicating the cytotoxic activity of Tri-T cells against leukemic cells *in vivo*. To observe infiltrated primary human leukemic cells, the BM of xenografted mice was analyzed by immunohistochemistry. Consistent with previous studies, we found that leukemia cell engraftment changed the morphology of mouse organs, including the enlargement of the spleen and the appearance of disseminating masses in the liver [46]. Hematoxylin staining of BM sections revealed compacted human AML blasts, distinguished from typical hematopoietic cells by their large size, round shape, and faint color. Moreover, in the BM of Tri-T cell-treated animals, infiltrating cells demonstrated an increased ratio of nucleus to cytoplasm, prominent nucleoli with high density typical of lymphocytes, and enhanced erythropoiesis (Figure 5D). Immunohistochemistry also revealed significantly larger subsets of human CD8^+^ cells in the BM of mice treated with Tri-T cell infusion, compared with untreated mice (Figure 5E), suggesting the effective elimination of human AML blasts by Tri-T cells *in vivo*.

## DISCUSSION

Because tumor cells continuously undergo genetic mutation, it is important to generate efficient and simultaneously activated multi-T cells without needing to know the exact epitopes. Multi-antigen specific T cells are vital to improve immunotherapy because these cells can overcome immune escape that results from tumor antigen loss. Previously, we addressed the importance of *WT1*, *survivin*, and *TERT* as LAAs. All AML patients express at least one of these three antigens [2, 5]. Therefore, this study investigated whether DCs simultaneously transfected with RNA encoding *WT1, survivin*, and *TERT* can induce multi-TAA-specific T cells, and we directly compared single-TAA-specific T cells and multi-TAA-specific T cells for each donor. The use of full RNA sequences for the antigens induced high affinity CTLs that recognized tumors, regardless of HLA type, and both CD4 and CD8 cells were produced, thus minimizing antigen escape by epitope loss variants. Tri-T cells generated by DCs with full TAA RNA were able to stimulate the production of both CD4^+^ effector cells and CD3^+^CD45RO^+^ memory cells in the population of CD62L^−^CCR7^−^ effector/memory cells, suggesting memory function. Further studies of Tri-T cell effects, including memory cell activity, will be required to develop efficient AML immunotherapy. *TERT* RNA-transfected human DCs can effectively stimulate CTLs and induce tumor cell lysis [29, 47]. DC vaccination has been shown to increase antigen presentation and production of effector T cells but not memory cells [48].

Our results are consistent with those of Weber et al [15]. and emphasize the need to generate multi-antigen-specific T cells that can be obtained in high quantities and used as an effective tool in cancer immunotherapy based on adoptive cell transfer. Theoretically, all of the T cells specific for the three antigens could present in the T cell repertoire. Therefore, the stimulation of the three antigens showed higher frequency of IFN-gamma secreting T cells compared to the stimulation with single antigen. When single T cells recognized WT1 as an dominant antigen and activated, cytokines released from this T cells and dendritic cells could provide a third signal for activation of the other T cells recognizing subdominant antigens such as TERT or surviving by modulating the TCR signaling threshold. IL-7 and IL-15 mediated TCR sensitization enables T cell responses to self-antigens such as tumor associated antigens [49]. IL-2 reduces the TCR threshold and IL-21 sustains CD28 expression on IL-15 activated naïve CD8 T cells [50, 51]. In study on the effect of inflammatory cytokines on naïve T cells, IL-1 increased CD4 T cells but the proliferation of CD8 T cells required IL-12 in response to antigen and IL-2 [52]. To treat lymphoma, CTLs targeting multiple tumor-associated antigens have been simultaneously generated *in vitro* by stimulation with DCs pulsed with overlapping peptide libraries including the targeted antigens [15, 37]. However, the combination of five different antigens in the TAA mix did not lead to inhibition of any single antigen response.

Interestingly, the T-cell response to tumors was higher in leukemic blasts that simultaneously expressed *WT1, survivin*, and *TERT* compared with leukemic blasts expressing any single antigen (Figure 4C). This suggests not only a way of overcoming immune escape but also an amplification of immunogenicity in response to multi-tumor antigen expression. Because T cells are activated by peptide epitopes when the antigens are bound to self-MHC molecules, MHC restriction and T cells from each donor can vary in terms of tumor antigen expression. Our data showed that *WT1* and *TERT* were predominantly produced in CD8+ and CD4+ T cells, respectively (Figure 3), but our study was limited by the number of donors and patients. This individual human data hints at strategies to further study tumor antigens. Further studies of correlation with HLA types must be conducted. Although human *WT1* and *survivin* have high amino acid sequence similarity with mouse antigens, syngeneic mouse models do not necessarily represent the activity of human antigen-specific T cells *in vivo*. Based on a previous study [47], we used a leukemia xenograft mouse model to examine anti-tumoral effects of Tri-T cells. Tri-T cells suppressed human blasts that expressed *WT1, survivin*, and *TERT* and remained in the BM. We will use the leukemia xenograft model to further investigate immunotherapeutic effects of Tri-T cells, including their combinations with anticancer drugs and immunomodulating agents, on overall survival of AML blasts [55]. AML is a heterogeneous disease with diverse LAAs and a great deal of antigen epitope variation. Because Tri-T cells can simultaneously target multi-LAA-expressing blasts, they are highly efficient against a complicated cancer. Tumors often recur after conventional T-cell therapy as a result of immune evasion through mutations in the genes encoding tumor antigens [53]. However, Tri-T cells can treat even multi-tumor antigen-expressing tumors.

In summary, we generated cost- and labor-effective Tri-T cells that can prevent AML recurrence. Multiple TAA-transfected DCs effectively induced production of multi-Tri T cells, resulting in the regression of leukemia, as demonstrated by low minimal residual blasts and engraftment in BM. Successful generation of triple antigen specific T cells and proper induction of Tri-T cell immune responses achieved in AML. Tri-T cells may be valuable for AML treatment.

## MATERIALS AND METHODS

### Human samples, mice and cell lines

Human peripheral blood was obtained from HLA-A*02:01 or HLA-A*24:02 healthy donors with the consent of the donors and approval from the Institutional Review Board of our institution. NOD/ShiLtSz-scid/IL2R_null (NOD.Cg-PrkdcscidIl2rgtm1Wjl/SzJ, termed NSG) mice were purchased from Jackson Laboratory and maintained in our animal facilities under pathogen-free conditions. Animal care and experiments were conducted according to our Institutional Animal Care and Use Committee guidelines. Cells were isolated using Ficoll-Paque™ PLUS (GE Healthcare Life Sciences, Piscataway, NJ, USA), followed by anti-CD14, anti-CD4 and anti-CD8 magnetic microbeads (Miltenyi Biotec, Bergisch Gladbach, Germany) according to the manufacturer's instructions. TF-1a and K562 cell lines were purchased from American Type Culture Collection (ATCC, Manassas, VA, USA).

### RNA electroporation into dendritic cells

To enhance proteasomal degradation and increase immune responses, we constructed antigen fusions, combining *WT1, survivin* and *TERT* with ubiquitin and ornithine decarboxylase in the pcDNA3 vector (Invitrogen, Grand Island, NY, USA) as previously described [30]. *In vitro* transcription and RNA electro-transfer into DCs were performed as previously described [15]. To achieve maturation, DCs were subsequently cultured in medium containing tumor necrosis factor alpha (10 ng/ml), IL-6 (10 ng/ml), IL-1*β* (10 ng/ml) (PeproTech) and prostaglandin E_2_ (1 μg/ml) (Sigma) for 24 hours.

### *In vitro* generation of leukemia-associated antigen-specific CD4 and CD8 T cells

LAA-specific CTLs were generated using autologous DCs as previously described [54]. Briefly, purified CD8 and Th1-polarized CD4 T cells were stimulated with *WT1*, *WT1*-*survivin* or *TERT* IVT mRNA-electroporated DCs. Seven days later, the cells were restimulated with RNA-electroporated DCs in medium containing 10 U/ml IL-2, 5 ng/ml IL-15. On day 20 after initial stimulation, the cells were harvested, and their phenotypes, specificity and functional capacity were analyzed. Cells were stained with PEcy5-CD4, FITC-CD8 and APC-Cy7-CD45RO (BD Pharmingen) and analyzed using a FACSCalibur flow cytometer (BD Biosciences, San Diego, CA, USA).

### RT-PCR for *WT1, survivin* and *TERT* mRNA

RNA extraction and RT-PCR analysis was performed as described previously [5]. The primers are as follows: for *WT1*, 5′-GCGGCGCAGTTCCCCAACCA-3′ (sense) and 5′ATGGTTTCTCACCAGTGTGCTT-3′ (antisense); for *survivin*, 5′- ATGGGTGCCCCGACGTTGCC-3′ (sense) and 5′- ATCCATGGCAGCCAGCTGCT-3′ (antisense); for *TERT*, 5′- GGGCCCGAATTCATGCGTCCCTGGGACACGCCTTG-3′ (sense) and 5′- GCTGTGCTGGCGGAGCAGAAACAGGGGCCG-3′ (antisense); for *Beta-actin*, 5′- TCACCCACACTGTGCCCAT-3′ (sense), 5′TCCTTAATGTCACGCACGATTT-3′ (antisense).

### ELISpot assay

To detect IFN-γ secretion, ELISpot assays were performed according to the manufacturer's instructions (552138, BD Bioscience). Briefly, T cells were serially diluted from 5 × 10^5^ to 5 × 10^4^ cells/well and then co-cultured with DCs that were either un-transfected or transfected with *WT1, survivin* and *TERT* mRNA in triplicate. The numbers of IFN-γ spots were analyzed using an AID-ELISpot-Reader (AID, Strassberg, Germany).

### Evaluation of Tri-T cell recognition of AML blasts

AML blasts were analyzed by RT-PCR for expression of *WT1, survivin* and *TERT*. Tri-T cells recognized HLA-A*02:01-partially matched AML blasts at 1:1 ratios (AML blasts: T cells) in the presence of IL-2 (25 U/ml). Un-stimulated T cells from the same donor were used as a control. On day three after co-culturing, CD3^+^ T cells and CD33^+^CD34^+^ residual AML blasts were detected by FACS. To detect IFN-γ production, ELISpot assays were performed according to the manufacturer's instructions (552138, BD Bioscience). Briefly, T cells were serially diluted from 5 × 10^5^ to 5 × 10^4^ cells/well and co-cultured with DCs that were either un-transfected or transfected with *WT1, survivin*, and *TERT* mRNA. This was performed in triplicate.

### Human xenograft model and adoptive transfer of Tri-T cells

All protocols for animal experiments were approved by the Institutional Animal Care and Use Committee of the Catholic University of Korea. For the xenograft model, NOD/ShiLtSz-scid/IL2R_null (NOD.Cg-PrkdcscidIl2rgtm1 Wjl/SzJ, termed NSG) mice were irradiated with 300 cGy prior to AML tumor cell injection. One day later, AML tumor cells were prepared at a final concentration of 1 × 10^7^ cells/200 μl PBS per mouse for intravenous injection. On days 20 and 27 after the injection of AML tumor cells, mice intravenously received Tri-T cells (1 × 10^7^ cells per mouse). Mice were analyzed for 15 weeks. Eight to 15 weeks after Tri-T cell injection, tissues were prepared to examine the function of the Tri-T cells.

### Histology and immunostaining

BM samples were fixed in paraformaldehyde (PFA), decalcified with 5% formic acid and embedded in paraffin. Prepared slides were counterstained with Meyer's hematoxylin. To confirm leukemic blasts in BM, hematoxylin and eosin (H&E) staining was used after fixation. For immunohistochemistry, BM samples were incubated with anti-human CD3 (Abcam, USA). All procedures were performed on a DAKO autostainer (DAKO Autostainer Plus, Dako, Glosrup, Denmark) at room temperature. Slides were counterstained with Meyer's hematoxylin. For fluorescent images, cells were fixed with 2% PFA for 10 min at room temperature. After washing with PBS, the cells were blocked with 5% horse serum and incubated with anti-human CD8 (Abcam, USA) and secondary antibodies. DAPI was used for nuclear staining, and the tissue was visualized under a fluorescence microscope (Carl Zeiss, Axiovert 200, Germany).

### Statistical analysis

All results are presented as the means ± SE. *P* values were calculated using paired student *t* tests with GraphPad Prism ver. 5 software (La Jolla, CA, USA). Values of P<0.05 were considered statistically significant.

## SUPPLEMENTARY MATERIALS FIGURE AND TABLE


