# Supplementary material for: Simultaneous in vitro generation of CD8 and CD4 T cells specific to three universal tumor associated antigens of WT1, survivin and TERT and adoptive T cell transfer for the treatment of acute myeloid leukemia
Source: Oncotarget. 2017 Apr 19;8(27):44059–72. doi: 10.18632/oncotarget.17212 (PMC5546462; doi:10.18632/oncotarget.17212)
Supplement: Supplementary file 1 [file oncotarget-08-44059-s001.pdf]

Simultaneous *in vitro* generation of CD8 and CD4 T cells specific to three universal tumor associated antigens of *WT1, survivin* and *TERT* and adoptive T cell transfer for the treatment of acute myeloid leukemia

SUPPLEMENTARY FIGURE AND TABLE

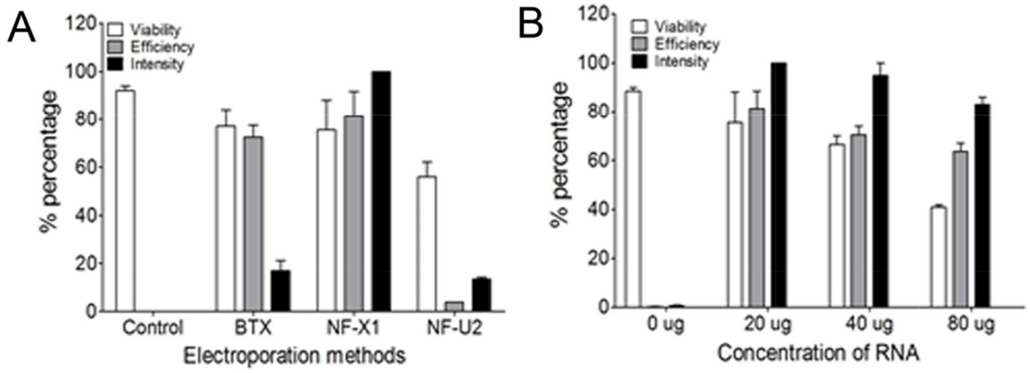

Supplementary Figure 1: (A) Comparison of electroporation methods for RNA. The optimal conditions for the different electroporation methods were selected and compared with respect to transfection efficiency, transfection intensity, cell viability after 48 hr. BTX electropration with 20 ug IVT RNA per 1 x 10^6 cells in 2 mm cuvettes at 300V/500 usec. Nucleofection (NF) with 10 ug pMax-GFP plasmid DNA per 1 x 10^6 cells, using programs X1, U2. **(B)** The optimal condition for IVT RNA concentration on nucleofector X1 program. DCs viability expressed as percentage of live DCs. Transfection efficiency expressed as percentage of DCs expressing GFP. Intensity calculated as a percentage of the strongest fluorescence achieved with the nucleofector program X1 with GFP IVT RNA.

Supplementary Table 1: HLA type of healthy donor

	HLA A	HLA A	HLA B	HLA B	HLA C	HLA C	HLA DR	HLA DR
N1	0201	0201	1301	3501	0303	0304	0803	0901
N2	0201	3001	1302	4801	0602	0801	0701	0701
N3	0201	1101	1501	5101	0401	1402	0406	1501
N4	0201	3303	3501	4403	0401	1403	1302	1501
N5	0201	0201	1511	4801	0303	0801	0901	1405
N6	0201	0206	1511	5101	0349	1402	0901	1501
N7	0201	2402	4001	5201	0304	1202	0403	1502
N8	0201	1101	1511	5401	0102	0303	0101	0405
N9	0201	2601	1501	4002	0303	0303	1201	1501
N10	0201	3303	4403	5401	0102	1403	0803	1302
N11	0201	1101	1501	4001	0304	0401	0406	0901
N12	0201	3001	1302	4001	0602	0702	1101	1302
N13	0206	3303	4403	5401	0102	1403	1302	1501
N14	0201	2601	1501	2705	0102	0401	0101	0901
